# Trabajo en equipo y desgaste profesional en trabajadores de la salud de un hospital público en Colombia

**DOI:** 10.7705/biomedica.7240

**Published:** 2025-03-28

**Authors:** Ana C. Amaya-Arias, Fabián Jaimes, Jenny García-Valencia

**Affiliations:** 1 Facultad Nacional de Salud Pública, Universidad de Antioquia, Medellín, Colombia Universidad de Antioquia Facultad Nacional de Salud Pública Universidad de Antioquia Medellín Colombia; 2 Facultad de Medicina, Universidad de Antioquia, Medellín, Colombia Universidad de Antioquia Facultad de Medicina Universidad de Antioquia Medellín Colombia

**Keywords:** agotamiento psicológico, agotamiento profesional, personal de salud, Burnout, psychological, burnout, professional, health personnel

## Abstract

**Introducción.:**

Se ha descrito una posible relación entre el trabajo en equipo y el desgaste profesional en el personal de salud. Las variables implicadas se asocian con la seguridad de los pacientes.

**Objetivo.:**

Determinar y describir la relación entre el trabajo en equipo y el desgaste profesional en trabajadores de la salud de un hospital de tercer nivel de Colombia.

**Materiales y métodos.:**

Se trata de un estudio analítico, observacional, de corte transversal, con un muestreo por conveniencia de 510 sujetos. Para evaluar el desgaste profesional se aplicaron los cuestionarios *TeamSTEPPS Teamwork Perceptions Questionnaire* y *Copenhagen Burnout Inventory;* se diligenció uno adicional para los datos demográficos y laborales. Los datos se recolectaron de forma digital. Se hizo un análisis descriptivo en el que se comparó el trabajo en equipo y el desgaste profesional entre unidades, por ocupación y según el promedio de horas laborales semanales. Finalmente, se calcularon matrices de correlaciones con los puntajes de ambas pruebas.

**Resultados.:**

Se encontraron puntuaciones de trabajo en equipo que se consideran moderadas a altas. El 31,4 % de los trabajadores reportó niveles moderados o altos de desgaste profesional. No se encontraron diferencias significativas por sexo o unidad de trabajo. Se encontró una correlación con fuerzas de asociación bajas a moderadas que muestran una relación inversa entre las subescalas del *TeamSTEPPS Teamwork Perceptions Questionnaire* y el *Copenhagen Burnout Inventory*.

**Conclusión.:**

El trabajo en equipo y el desgaste profesional pueden tener una relación inversa, en la que un mejor trabajo en equipo resulta en una percepción menor de desgaste en los trabajadores de la salud. Los resultados de este estudio deben interpretarse con precaución debido a posibles sesgos.

## Abstract (en)

**Introduction.:**

Previous reports have described a relationship between teamwork and burnout in health workers, involving variables associated with patients’ safety.

**Objective.:**

To determine and describe the relationship between teamwork and burnout perceived by health workers from a tertiary-level hospital in Colombia.

**Materials and methods.:**

We conducted a cross-sectional, observational, analytical study with a convenience sample of 510 participants. We applied the TeamSTEPPS Teamwork Perceptions Questionnaire and the Copenhagen Burnout Inventory to assess burnout. An additional survey was conducted to collect demographic and employment data. Data was collected digitally. Descriptive analyses were carried out to compare teamwork and burnout across units, by occupation, and according to the average number of hours worked per week. Finally, correlation matrices were calculated with the scores from both tests.

**Results.:**

Teamwork levels were moderate to high, and 31.4% of workers reported moderate or high levels of burnout. No significant differences were found based on sex or work unit. Results from the TeamSTEPPS Teamwork Perceptions Questionnaire and the Copenhagen Burnout Inventory were correlated with a low and moderate strength of association, showing an inverse relationship between them.

**Conclusion.:**

Teamwork and burnout may have an inverse relationship, where better teamwork results in a lower perception of burnout among healthcare workers. However, the results of this study should be interpreted with caution due to potential biases.

Los eventos adversos son considerados un problema de salud pública, ya que son una de las principales causas de muerte en el mundo ^1^. La mejor forma de abordar esta problemática es mediante la prevención, lo que requiere conocer la incidencia y los factores asociados con la ocurrencia de dichos eventos adversos.

Una revisión de alcance, que incluyó estudios de diferentes países entre 1997 y 2018, encontró que la mediana de las incidencias de eventos adversos era del 10 % ^2^, dato congruente con la incidencia reportada en otros estudios ^3^^,^^4^. Estos hallazgos confirman que el panorama de la seguridad del paciente se ha mantenido más o menos estable a lo largo de las últimas décadas, a pesar de los esfuerzos y el gran número de investigaciones realizadas en esta área ^5^.

En la última década, un creciente número de investigaciones se ha enfocado en el reconocimiento y el análisis de algunos factores humanos -como las habilidades de trabajo en equipo y el bienestar laboral- y su impacto en el rendimiento de los trabajadores de la salud, la calidad de los servicios sanitarios y la seguridad de los pacientes ^6^^-^^8^. Algunos estudios relacionados con el tema han demostrado que al implementar estrategias para fortalecer la cultura de la seguridad y las habilidades de trabajo en equipo, se evidencia una mejoría en la sensación de bienestar y en la resiliencia de los trabajadores de la salud, se reducen el riesgo de desgaste profesional (*burnout*) y los errores en la atención ^7^^,^^9^^-^^12^.

La propuesta que subyace la comprensión de todos esos factores resalta la importancia de reconocer la actividad de los equipos de salud desde un “enfoque sistémico”, en el que todos los componentes hacen parte del sistema como un conjunto, en lugar de unidades independientes ^13^. En dicho sistema se relacionan tres factores principales:

1. las habilidades clínicas individuales;
2. el trabajo en equipo entre los equipos y dentro de ellos, y
3. el entorno, que incluye aspectos físicos como el ruido y humanos como la presión del tiempo y la sobrecarga laboral ^14^.

Esto ha llevado a que se adelanten investigaciones para evaluar la relación entre las habilidades de trabajo en equipo, como la comunicación o el liderazgo, y el desgaste profesional ^15^^-^^18^.

En varios de estos estudios se ha encontrado una asociación entre las variables mencionadas. Los resultados demuestran que en aquellas unidades o instituciones donde se manejan mejores estrategias de trabajo en equipo, el estrés laboral y el riesgo de desgaste profesional se reducen y que, a peor liderazgo o comunicación, mayor es el agotamiento ^19^^,^^20^. Además, autores como Wang *et al.*^21^ han encontrado que la relación puede ser bidireccional. En su estudio, el desgaste laboral tuvo un efecto negativo en la forma en la que los trabajadores se relacionaban y en su desempeño. Aquellos con mayor nivel de agotamiento presentaban comportamientos que generaban cargas adicionales para los colegas, como retrasos en la ejecución de las tareas o interacciones más estresantes con ellos.

El desgaste profesional se caracteriza por cansancio o agotamiento emocional, distanciamiento y sensación de falta de realización personal ^22^. Por su parte, el distanciamiento se define como una estrategia de afrontamiento que usa una persona para poner distancia entre sí misma y los sujetos con los que trabaja, lo que lleva a los trabajadores a mostrar indiferencia o una actitud cínica con los demás. Desde lo teórico, podría haber una relación entre estar en estado de agotamiento y tener una menor capacidad para trabajar en equipo de forma efectiva.

En Colombia, aunque se han realizado investigaciones para evaluar el desgaste profesional en los trabajadores de la salud, solo algunos proyectos de grado de enfermería han buscado evaluar su relación con el trabajo en equipo, uno de ellos con una población de estudio pequeña y solo en la unidad de cuidados intensivos ^23^ y el otro es una revisión de tema ^24^.

El realizar investigaciones en este campo de estudio puede permitir comprender mejor las dinámicas entre el trabajo en equipo y el desgaste profesional. Esto, a su vez, podría facilitar esfuerzos para mejorar el trabajo en equipo en las instituciones de salud, lo que podría aumentar el bienestar de los trabajadores, reducir su riesgo de desgaste profesional e impactar positivamente la calidad de los servicios prestados y la seguridad de los pacientes.

Teniendo en cuenta estos antecedentes, el objetivo de esta investigación fue determinar la relación entre el trabajo en equipo y el desgaste profesional en los trabajadores de la salud de un hospital de tercer nivel de Colombia.

## Materiales y métodos

### Diseño

Se llevó a cabo un estudio analítico, observacional, de corte transversal, en un hospital público, descentralizado, de alta complejidad, ubicado en una ciudad intermedia del sur de Colombia. Se incluyó personal asistencial de todas las unidades, ocupaciones, edades y sexos. El hospital contaba con 1.438 trabajadores asistenciales: 727 (50,6 %) auxiliares de enfermería, 220 (15,3 %) enfermeros, 36 (2,5 %) médicos generales, 214 (14,9 %) especialistas, 109 (7,6 %) subespecialistas y 132 (9,2 %) de otros profesionales de la salud. La recolección de datos se realizó del 1° de noviembre al 31 de diciembre de 2022.

### Muestra

Se realizó un muestreo por conveniencia con los siguientes criterios de inclusión: trabajadores de la salud que brindaron atención asistencial en el hospital durante el periodo de toma de datos y que aceptaron participar voluntariamente. Se excluyeron los trabajadores administrativos. Se esperaba lograr una participación del 80 % (1.150 individuos) para obtener un diagnóstico representativo, y la recolección de datos se completó al finalizar el periodo de medición debido a la voluntariedad de la participación. No se realizó muestreo aleatorio o estratificado dadas las limitaciones para acceder a las listas del personal, aspecto acordado con el comité de ética.

### Variables

*Trabajo en equipo:* actividades realizadas por equipos de dos o más individuos, con roles definidos aunque interdependientes, que cuentan con funciones y tareas específicas, interactúan y se coordinan para lograr objetivos comunes ^25^. Entre las habilidades fundamentales para el trabajo en equipo en el entorno clínico están la comunicación, el liderazgo, el apoyo mutuo y la conciencia situacional ^26^^,^^27^.

*Desgaste profesional:* serie de respuestas negativas hacia el trabajo, generadas por una exposición prolongada a un ambiente laboral altamente demandante y caracterizadas por cansancio o agotamiento emocional, distanciamiento o cinismo y sensación de falta de realización personal ^22^.

### Instrumentos de medición

El *TeamSTEPPS Teamwork Perceptions Questionnaire - Spanish* (T-TPQ-S) consta de 36 ítems que evalúan con una escala Likert bipolar de cinco puntos (1: totalmente en desacuerdo, 5: totalmente de acuerdo), la percepción que las personas tienen sobre cinco dimensiones del trabajo en equipo en su unidad: estructura del equipo ^7^, liderazgo ^7^, conciencia situacional ^7^, apoyo ^7^ y comunicación ^8^. Este cuestionario tiene estudios previos en inglés de validez y confiabilidad ^28^, y cuenta con validación para Colombia en la cual se incluyó un nuevo ítem en la subescala de comunicación. Esta inclusión resultó en índices adecuados de consistencia interna, valores de alfa de Cronbach que oscilan entre 0,848 y 0,916, y un ajuste adecuado del análisis factorial confirmatorio al modelo teórico del instrumento ^29^. La versión en español se presenta en el anexo 1.

*Copenhagen Burnout Inventory* (CBI) cuenta con 19 ítems que evalúan por medio de una escala Likert bipolar de cinco puntos (0: nunca, 4: siempre), la percepción que los trabajadores tienen sobre su nivel de cansancio o desgaste profesional. Consta de tres subescalas: desgaste personal ^6^, desgaste relacionado con el trabajo ^7^ y desgaste relacionado con los usuarios ^6^. El instrumento fue validado en español por Molinero Ruiz *et al.*^30^, con indicadores adecuados de confiabilidad y validez de constructo estructural.

Además, se aplicó un cuestionario para recolectar los siguientes datos demográficos de los participantes: sexo, ocupación, edad, unidad o servicio, tipo de vinculación laboral, tiempo de experiencia en la ocupación actual, tiempo laborando en la institución, tipo de contratación y horas promedio de trabajo a la semana.

### Recolección de los datos

Los datos se recolectaron de forma digital en un formulario de Google Forms. La investigadora principal visitó cada uno de los servicios y presentó a los trabajadores los objetivos del estudio, reforzó el carácter confidencial de todos los datos por recolectar y resolvió las dudas. Finalmente, a quienes aceptaban participar, se les enviaba el enlace de acceso al consentimiento informado que, una vez firmado, llevaba de forma automática a los formularios de los instrumentos. El enlace de acceso fue enviado de forma masiva por correo electrónico y a los grupos de WhatsApp de cada servicio, una vez realizadas las visitas de difusión.

### Análisis de los datos

Se calcularon los puntajes totales y por subescala de cada prueba. Para el T-TPQ-S se siguieron las recomendaciones dadas por los autores, en las cuales la puntuación para cada dimensión del trabajo en equipo se calculó sumando las puntuaciones de cada ítem y el puntaje total se calculó sumando las puntuaciones de las cinco subescalas ^28^. Para el caso del CBI, los puntajes totales se calcularon siguiendo las indicaciones dadas por Molinero Ruiz *et al.*^30^: se convirtieron las puntuaciones directas a valores normalizados, donde 0 correspondía a 0, 1 a 25, 2 a 50, 3 a 75 y 4 a 100. Con estos valores se calculó el valor promedio de las puntuaciones por subescala y el promedio de todos los ítems se tomó como el valor total de la prueba.

Se evaluó si las variables cuantitativas cumplían el supuesto de normalidad, mediante la prueba de Shapiro-Wilk. Se encontró que ninguna variable tenía una distribución normal, por lo tanto, todos los análisis estadísticos realizados fueron no paramétricos.

Se calcularon las medidas de tendencia central y de dispersión de las dimensiones del trabajo en equipo y del desgaste profesional de la institución. Además, los puntajes totales de las pruebas se categorizaron teniendo en cuenta las recomendaciones de los autores de cada instrumento, quienes definieron los puntos de corte categorizando los niveles percibidos de trabajo en equipo y desgaste profesional así:

- T-TPQ-S: los valores de cuatro y cinco se consideraron altos en cuanto a la medición de la habilidad y los valores iguales o menores de tres se tomaron como normales a bajos ^28^.
- CBI: tanto para las subescalas como para el puntaje total, los rangos establecidos fueron: alto (≥ 75), moderado (50-74,9) y leve (< 50). Estas categorías se definieron considerando que los puntajes se modificaron a valores normalizados según las recomendaciones de los autores ^31^.

Para las variables cualitativas se construyeron tablas de frecuencias y se calcularon los porcentajes. Se hicieron comparaciones de las dimensiones del trabajo en equipo y el desgaste profesional entre unidades, por ocupación y según el promedio de horas laboradas por semana. Para esto se usó la prueba H de Kruskal-Wallis, se calcularon los tamaños del efecto con el coeficiente eta al cuadrado , también llamado razón de correlación, que representa el porcentaje de la varianza de la variable dependiente explicado por la variable independiente. Un coeficiente eta menor de 0,010 se considera un efecto trivial; entre 0,010 y 0,059, un efecto pequeño; entre 0,060 y 0,139, mediano; y mayor o igual de 0,14, un efecto grande ^32^. De igual forma, se compararon las puntuaciones de cada prueba por sexo y afiliación laboral adicional o empleo adicional en otra institución. En este caso se usó la prueba U de Mann-Whitney. Los tamaños del efecto se calcularon con la correlación biserial puntual.

Dado que se realizaron múltiples comparaciones y con la intención de reducir el riesgo de falsos positivos, se aplicó la corrección de Benjamini- Hochberg ^33^. Estos valores de p, corregidos, se tuvieron en cuenta para determinar la significancia estadística. En todos los casos se calcularon los intervalos de confianza.

Se calcularon matrices de correlaciones con los puntajes de ambas pruebas para determinar la relación entre las dimensiones del desgaste profesional con las dimensiones del trabajo en equipo. Para esto, se calcularon correlaciones de Spearman, cuya corrección de los valores de p se hizo mediante el método de Sidak ^34^. Todos los análisis se ejecutaron en el *software* Stata™, versión 15.

### Consideraciones éticas

Este proyecto cumple con las normas nacionales e internacionales de investigación en humanos, incluida la Declaración de Helsinki ^35^ y la Resolución 8430 de 1993 ^36^; fue aprobado por los comités de ética e investigación del hospital participante (acta de aprobación 006-002 del 28 de julio del 2020) y de la Facultad Nacional de Salud Pública de la Universidad de Antioquia (acta de sesión No. 228 del 21 de febrero del 2020). Dado que la aplicación de los instrumentos se hizo de forma digital, la toma del consentimiento informado también se hizo en este formato, como se explica en el apartado de recolección de datos.

## Resultados

### Descripción de la muestra

De 1.150 sujetos, se lograron recolectar datos de 510, que correspondían a la población objetivo. En el cuadro 1 se resumen las principales características de los participantes.

**Cuadro 1.** Características demográficas del personal asistencial que participó en del estudio

Variable	Categoría	n	%
Sexo	Hombre	143	28
	Mujer	367	72
Edad	18 a 26	83	16,3
	27 a 59	423	82,9
	> 60	4	0,8
Ocupación	Auxiliar de enfermería	228	44,7
	Enfermero	79	15,5
	Médico general	53	10,4
	Médico especialista o subespecialista	63	12,4
	Otra profesión asistencial	87	17,0
Unidad	Hospitalización	184	36,0
	Hospitalización y sala de partos	23	4,5
	Unidad de cirugía	74	14,5
	Urgencias	76	14,9
	Unidad de cuidados intensivos	31	6,1
	Otras	122	23,9
Tiempo de experiencia laboral	< 6 meses	18	3,5
	Entre 6 meses y 1 año	30	5,9
	Entre 1 y 3 años	83	16,3
	Entre 3 y 5 años	44	8,6
	Más de 5 años	335	65,7
Tiempo laborando en el hospital	< 6 meses	70	13,7
	Entre 6 meses y 1 año	67	13,1
	Entre 1 y 3 años	121	23,7
	Entre 3 y 5 años	73	14,3
	Más de 5 años	179	35,1
Tipo de contrato	Agremiación	379	74,3
	Prestación de servicios	13	2,6
	Contrato de planta	93	18,2
	Contratación mixta	4	0,8
	Interno en rotación	21	4,1
Labora en otra entidad	Sí	100	19,6
	No	410	80,4
Horas de trabajo semanal	< 40	27	5,3
	40 a 59	293	57,5
	60 a 79	119	23,3
	> 80	71	13,9

El grupo de trabajadores de la salud que hizo parte del estudio estuvo conformado principalmente por mujeres (72 %), en su mayoría auxiliares de enfermería (44,7 %), especialmente de las unidades de hospitalización (36 %). La muestra refleja proporcionalmente la distribución ocupacional del hospital, el 65,7 % de los trabajadores cuenta con más de cinco años de ejercicio profesional lo cual implica una amplia experiencia en su campo; el 73,1 % del grupo lleva laborando en el hospital más de un año. El principal tipo de contratación del grupo fue por agremiación (74,3 %), la mayoría trabaja solo en el hospital (80,4 %) y el tiempo promedio más frecuente de horas laboradas por semana fue entre 40 y 59 horas (57,5 %), es decir, tiempo completo. En cuanto a la edad, la mediana fue de 36 años (RIQ = 29-43), con un mínimo de 18 y un máximo de 65 años. La mayor parte del grupo (82,9 %) estuvo en el rango de adultez (27 a 59 años).

### Trabajo en equipo

En el cuadro 2 se presenta el resumen de los resultados obtenidos en la prueba T-TPQ-S. Aunque en todas las subescalas se obtuvieron valores en todo el rango posible, las medianas calculadas en las subescalas de la prueba reflejan puntuaciones de trabajo en equipo consideradas moderadas a altas. En cuanto al puntaje total, se encontró una mediana de tres (RIQ = 3-4; mínimo = 1; máximo = 5), teniendo en cuenta que el rango de puntuaciones de esta prueba va desde uno hasta cinco, en general, la percepción del trabajo en equipo en el hospital participante se considera entre moderada y alta.

**Cuadro 2:** Puntajes obtenidos en la prueba *TeamSTEPPS Teamwork Perceptions Questionnaire* en el hospital

Subescala	Descripción	Mediana (RIQ)	Puntaje alto	n	%
Estructura del equipo	Forma en que el personal se estructura y organiza de manera efectiva como equipo al compartir objetivos comunes, comunicar planes y coordinar acciones para su logro.	4	No	239	46,9
		(3,6-4,1)	Sí	271	53,1
Liderazgo	Entrega de instrucciones, asertividad y apoyo Gestión adecuada de recursos para evitar el malgasto o la sobrecarga de algún miembro del equipo y contar con lo necesario de forma oportuna Ejemplo y motivación para la excelencia	3,85	No	291	57,1
		(3,4-4)	Sí	219	42,9
Conciencia situacional	Observación del equipo y conciencia de los procesos en curso para apoyar tareas Anticipación y corrección de errores, incluso antes de que estos ocurran	3,85	No	282	55,3
		(3,3-4)	Sí	228	44,7
Apoyo mutuo	Ayuda mutua entre los integrantes del equipo para la ejecución de tareas, retroalimentación positiva y solución de conflictos.	3,71	No	330	64,7
		(3,3-4)	Sí	180	35,3
Comunicación	Calidad, cantidad y oportunidad de la información intercambiada entre los miembros del equipo. Asertividad en el intercambio de dicha información	4	No	227	44,5
		(3,6-4,1)	Sí	283	55,5
Puntaje total	Percepción general del trabajo en equipo en la unidad	139	No	349	68,4
		(126-146)	Sí	161	31,6

RIQ: rango intercuartílico

No se encontraron diferencias estadísticamente significativas en la medición del trabajo en equipo o sus dimensiones por sexo, unidad, ocupación o empleo adicional en otra institución. Los tamaños del efecto muestran que ninguna de estas variables se asoció con la medición del trabajo en equipo.

En el cuadro 3 se evidencia una diferencia estadísticamente significativa entre las horas de trabajo semanales y las medianas de las subescalas “estructura del equipo” y “comunicación”. Sin embargo, la magnitud de dicho efecto es bastante baja y no significativa para el caso de la subescala de “comunicación”.

**Cuadro 3.** Comparación de los puntajes obtenidos en la prueba *TeamSTEPPS Teamwork Perceptions Questionnaire* según la cantidad de horas laborales semanales

Subescala	Menos de 40 horas	40-59 horas	60-79 horas	80 horas o más	p^1^	p BH^2^
	Mediana (RIQ)	Mediana (RIQ)	Mediana (RIQ)	Mediana (RIQ)		
Estructura del equipo	4 (3,4-4,1)	3,8 (3,5-4,1)	4 (3,7-4,4)	4 (3,7-4,2)	0,002	0,006*
Liderazgo	3,8 (3,1-4,4)	3,8 (3,2-4)	3,8 (3,4-4,1)	4 (3,5-4,3)	0,530	0,530
Conciencia situacional	3,8 (3,1-4,1)	3,8 (3,2-4)	4 (3,5-4)	3,8 (3,4-4)	0,351	0,421
Apoyo mutuo	3,5 (3,1-4)	3,7 (3,3-4)	3,8 (3,4-4)	3,8 (3,6-4)	0,135	0,270
Comunicación	4 (3,8-4)	3,8 (3,6-4)	4 (3,6-4,4)	4 (3,7-4,3)	0,001	0,006*
Total	3 (3-4)	3 (3-4)	3 (3-4)	3 (3-4)	0,320	0,421

^1^ Prueba H de Kruskal-Wallis

^2^ Valor ajustado de p de Benjamini-Hochberg

^3^ Coeficiente eta al cuadrado

^4^ IC: intervalo de confianza del n^2^

*p ≤ 0,05

### Desgaste profesional

En el cuadro 4 se presenta el resumen de los resultados obtenidos en la prueba CBI. En la medición del desgaste profesional, las respuestas de los sujetos cubrían todo el rango posible de la escala, pero fue mayor el porcentaje de los que manifestaron valores bajos a moderados de agotamiento. En cuanto al puntaje total, la mediana fue de 31,5 (RIQ = 2143,4; mínimo = 0; máximo = 100). De las tres subescalas, aquella con mayor porcentaje de sujetos con agotamiento moderado o alto fue la de desgaste personal (38,8 %). Del total de la muestra, el 17,5% de las respuestas también indicaron niveles moderados o altos de desgaste profesional.

**Cuadro 4:** Puntajes obtenidos en la prueba *Copenhagen Burnout Inventory*

Subescala	Descripción	Mediana (RIQ)	Rangos	n	%
Desgaste personal	Grado de fatiga o agotamiento emocional que experimenta la persona a nivel general	38	Bajo	353	69,2
		(21-50)	Moderado	139	27,3
			Alto	18	3,5
Desgaste relacionado con el trabajo	Agotamiento que experimenta la persona asociada con el trabajo, sin pretender establecer relaciones causales	31	Bajo	398	78,1
		(21-46)	Moderado	94	18,4
			Alto	18	3,5
Desgaste relacionado con los usuarios	Grado de fatiga o agotamiento que experimenta la persona asociada con su trabajo con otras personas	25	Bajo	415	81,3
		(13-42)	Moderado	81	15,9
			Alto	14	2,8
Total	Nivel de percepción general de agotamiento emocional	31,5	Bajo	421	82,5
		(21-43,4)	Moderado	81	15,9
			Alto	8	1,6

No se encontraron diferencias en las puntuaciones del CBI por sexo, unidad u horas de trabajo a la semana. Los tamaños del efecto demuestran que ninguna de estas variables influyó en la percepción del agotamiento de los trabadores del hospital.

Se encontró una diferencia estadísticamente significativa y con efecto pequeño entre las medianas de las subescalas “desgaste personal” y “desgaste relacionado con el trabajo”, en la catergoría de ocupación; y entre las medianas del “desgaste relacionado con el trabajo” y tener un empleo adicional en otra institución. Estas diferencias se ilustran en los cuadros 5 y 6.

**Cuadro 5.** Comparación de los puntajes obtenidos en la prueba *Copenhagen Burnout Inventory*, por ocupación

Subescala	Auxiliar de enfermería	Enfermero(a)	Médico(a) general	Especialista	Otra profesión asistencial	p1
	Mediana (RIQ)	Mediana (RIQ)	Mediana (RIQ)	Mediana (RIQ)	Mediana (RIQ)	
Desgaste personal	33	46	42	46	33	0,001
	(21-46)	(29-54)	(33-54)	(25-54)	(17-46)	
Desgaste trabajo	29	36	39	36	29	0,000
	(18-43)	(21-54)	(29-50)	(25-50)	(18-39)	
Desgaste usuarios	25	25	38	29	25	0,087
	(8-42)	(13-46)	(25-50)	(17-46)	(13-35)	
Total	32,8	32,8	31,5	31,5	27,6	0,640
	(22,3-44,7)	(23,6-46)	(19,7-40,7)	(20,8-40,7)	(17,1-42,1)	

RIQ: rango intercuartílico

^1^ Prueba H de Kruskal-Wallis

^2^ Valor ajustado de p de Benjamini-Hochberg

^3^ Coeficiente eta al cuadrado (n^2^)

^4^ IC: intervalo de confianza de n^2^

*p ≤ 0,05

**Cuadro 6.** Comparación de los puntajes obtenidos en la prueba *Copenhagen Burnout Inventory* para la variable “trabaja en otra institución”

Subescala	Trabaja en otra institución		p1	p BH^2^	***rbis*3**	IC _95%_^4^
	No Mediana (RIQ)	Sí Mediana (RIQ)				
Desgaste personal	35 (21-50)	42 (25-52)	0,110	0.22	-0,059	(-0,144 - 0,027)
Desgaste por trabajo	29 (21-43)	36 (25-54)	0,005	0.02	-0,118	(-0,202 - -0,032)
Desgaste por usuarios	25 (13-42)	29 (13-42)	0,640	0.64	-0,028	(-0,114 - 0,058)
TOTAL	31,5 (19,7-43,4)	32,2 (23,6-46)	0,197	0.262	-0,051	(-0,137 - 0,035)

^1^ Prueba U de Mann-Whitney

^2^ Valor ajustado de p de Benjamini-Hochberg

^3^ Correlación biserial puntual

^4^ IC: intervalo de confianza de la correlación biserial puntual

### Asociación entre trabajo en equipo y desgaste professional

Se encontraron fuerzas de asociación bajas y moderadas que demuestran una relación inversa entre los puntajes de las pruebas T-TPQ-S y CBI y sus subescalas. Las correlaciones oscilaron entre -0,0420 y -0,3309. Los resultados entre todas las subescalas se presentan en el cuadro 7.

**Cuadro 7.** Correlación de los resultados del TeamSTEPPS Teamwork Perceptions Questionnaire y el Copenhagen Burnout Inventory

Subescala	Coeficiente de correlación de Spearman (IC1)			
	Desgaste personal	Desgaste por el trabajo	Desgaste por los usuarios	CBI (puntaje total)
Estructura del equipo	-0,3246* (-0,400 - -0,245)	-0,3169* (-0,393 - -0,237)	-0,2932* (-0,371 - -0,212)	-0,0521 (-0,138 - 0,035)
Liderazgo	-0,2319* (-0,312--0,148)	-0,2669* (-0,346--0,184)	-0,2664* (-0,307--0,142)	-0,0306 (-0,117-0,056)
Conciencia situacional	-0,2942* (-0,372--0,213)	-0,3157* (-0,392--0,235)	-0,2961* (-0,373--0,215)	-0,0751 (-0,161-0,012)
Apoyo mutuo	-0,2722* (-0,351--0,190)	-0,2786* (-0,357--0,197)	-0,2486* (-0,328--0,165)	-0,0502 (-0,136-0,037)
Comunicación	-0,2692* (-0,348--0,187)	-0,2966* (-0,374--0,215)	-0,2809* (-0,359--0,199)	-0,0420 (-0,128-0,045)
T-TPQ-S (puntaje total)	-0,3061* (-0,383--0,225)	-0,3309* (-0,406--0,251)	-0,2956* (-0,373--0,214)	-0,0671 (-0,153-0,020)

T-TPQ-S: *TeamSTEPPS Teamwork Perceptions Questionnaire*; CBI: *Copenhagen Burnout Inventory*

1 IC: intervalos de confianza

* p ≤ 0,05 luego de la corrección de Sidak

Como se observa en los resultados, todas las subescalas del T-TPQ-S tuvieron correlaciones estadísticamente significativas con las subescalas del CBI, la mayoría mostró una fuerza de asociación baja. Se encontraron fuerzas de asociación moderadas e inversas entre las subescalas “estructura del equipo” con “desgaste personal” y “desgaste relacionado con el trabajo”, y entre la “conciencia situacional” y el “desgaste relacionado con el trabajo”. El puntaje total del T-TPQ-S se correlacionó de forma moderada e inversa con las tres subescalas del CBI, pero no son el puntaje total de esta prueba. El puntaje total del CBI no se correlacionó con ninguna de las subescalas del T-TPQ-S.

## Discusión

Los resultados reflejan una percepción favorable del trabajo en equipo en el hospital, en un rango moderado a alto (mediana =3; RIC = 3-4). Los puntajes de las subescalas tuvieron medianas entre 3,71 (apoyo mutuo) y 4,0 (estructura del equipo y comunicación), que reflejan niveles aceptables a buenos. Estos hallazgos son coherentes con otros estudios en los que se ha aplicado el T-TPQ en personal de salud, donde generalmente se observan puntuaciones intermedias a altas ^37^^-^^39^. Incluso en Colombia, cuando se ha medido el trabajo en equipo en entornos quirúrgicos con el instrumento *Observational Teamwork Assessment for Surgery* en español (OTAS-S), las mediciones antes de una intervención se ubicaban en estos mismos rangos (moderado y moderado-alto) ^40^. Esto sugiere que, en general, los trabajadores de la salud perciben que cuentan con habilidades adecuadas de trabajo en equipo, lo que facilita la colaboración efectiva y el logro de objetivos compartidos. Esto demuestra, además, que hay poca probabilidad de que en la medición del trabajo en equipo en este estudio se haya presentado un sesgo.

En cuanto al desgaste profesional se observa una predominancia de puntajes bajos que denotan estados leves de agotamiento (mediana = 31,5; RIQ = 21-43,4). Se resalta que el desgaste relacionado con los usuarios registra el puntaje más bajo (mediana = 24; RIQ = 13-42), en contraste con un puntaje más elevado de desgaste *t* personal (mediana = 38; RIQ = 21-50). Al desglosar estos datos, se encuentra que el 30,8 % de los participantes reportaron niveles moderados o altos de desgaste personal, mientras que el 21,9 y el 18,7 % indicaron niveles similares para el desgaste relacionado con el trabajo y con los usuarios, respectivamente.

Al comparar estos hallazgos con la literatura existente, se observa una tendencia interesante: una revisión sistemática de Alahmari *et al.* incluyó 41 estudios (13 de los cuales utilizaron el CBI) y encontró que, en promedio, los trabajadores de la salud reportaron niveles de agotamiento personal, relacionado con el trabajo y relacionado con los usuarios del 43, el 34 y el 38 %, respectivamente ^41^. Otros estudios, como el de Creedy *et al* reportaron medias aún más altas de desgaste personal y relacionado con el trabajo, especialmente entre las parteras (personal = 55,9 %; relacionado con el trabajo = 44,69 % y relacionado con los usuarios = 19,32 %) ^31^. Además, investigaciones adicionales han señalado prevalencias de agotamiento significativamente altas en distintos grupos de trabajadores de la salud. Por ejemplo, Majeed *et al.* encontraron que más del 50 % de los residentes evaluados tenía algún grado de agotamiento ^42^; mientras que Ilic *et al.* encontraron valores aún más altos al medir el desgaste en médicos y enfermeras de urgencias. El nivel de desgaste personal fue elevado para el grupo de médicos (media = 62,08 ± 18,08) y para el de enfermeras (media = 56,00 ± 17,55), al igual que el relacionado con el trabajo (médicos = 57,76 ± 20,57; enfermeras = 46.39 ± 24.01) y con usuarios (médicos = 62,31 ± 22,1; enfermeras = 51,93 ± 26,25) ^43^.

En este contexto, los resultados del presente estudio podrían indicar una de dos posibilidades: una dinámica particular en el hospital evaluado, donde los trabajadores de salud mantienen una relación más saludable con los usuarios, pero experimentan niveles más altos de agotamiento personal; o una renuencia personal para reportar el desgaste que se asocia específicamente con la atención a los pacientes. Por lo tanto, es importante considerar la posibilidad de un sesgo en la medición del desgaste en nuestro estudio, una cuestión que se abordará con mayor detalle en la sección de limitaciones. Esta discrepancia con la literatura existente subraya la necesidad de una exploración más profunda del trabajo en equipo en la institución para comprender mejor las dinámicas subyacentes y los factores que podrían influir en el reporte de los niveles reales de agotamiento.

En nuestro estudio, no se observaron diferencias significativas -ni desde la perspectiva estadística ni desde la clínica- en las mediciones de trabajo en equipo y el desgaste en función del sexo o la unidad. No obstante, se evidenció que la ocupación y el trabajar en otra institución influían la percepción del desgaste relacionado con el trabajo. Este hallazgo concuerda con investigaciones previas que han señalado que las jornadas laborales extendidas o las dobles jornadas pueden resultar en un incremento del agotamiento, a nivel agudo y crónico, lo que a su vez puede tener un impacto negativo en la seguridad del paciente ^44^.

Estudios anteriores han demostrado que dormir menos de seis horas por día está asociado con tasas más altas de errores en la atención (c2 = 4,34, RR = 1,3), así como trabajar más de 70 horas a la semana (c2 = 8,74, RR = 1,5). Además, es común que los profesionales de la salud trabajen turnos largos o nocturnos, lo que puede llevar a trastornos del sueño y a un descanso de baja calidad, incluso cuando no están de servicio. Esta dinámica puede traducirse gradualmente en fatiga crónica y afecta no solo las funciones ejecutivas sino también la empatía, exacerbando las tensiones y los factores estresantes en el lugar de trabajo ^45^. En este sentido, Abhiram *et al.* argumentan que “los turnos que duran ocho horas o menos pueden proteger contra el agotamiento y subrayan la importancia de los ciclos de trabajo y descanso adecuados y la eliminación de turnos prolongados como una estrategia potencial para prevenir el agotamiento” ^46^. Por lo tanto, sugieren la regulación de las condiciones laborales de los trabajadores de la salud mediante contratos de exclusividad, bien remunerados, de modo que se puedan controlar las jornadas laborales y establecer un límite máximo de horas semanales de trabajo.

Las diferencias del presente estudio encontradas por ocupación concuerdan con los hallazgos de Ilic *et al*., quienes identificaron diferencias significativas en la percepción del desgaste relacionado con el trabajo y con los usuarios entre médicos y enfermeras ^43^. En este caso, las enfermeras y los especialistas tuvieron medianas de agotamiento más altas en comparación con los auxiliares de enfermería y otros profesionales asistenciales. Finalmente, los resultados de este estudio son consistentes con los de Creedy *et al*. ^31^, quienes no encontraron diferencias significativas en las mediciones de desgaste en función del sexo y de otros factores individuales evaluados. Estos hallazgos señalan una complejidad subyacente en las dinámicas de desgaste y trabajo en equipo en el personal de salud, lo que resalta la necesidad de que en investigaciones futuras se pueda explorar más profundamente cómo estas variables podrían estar influenciadas por aspectos individuales u organizacionales.

El objetivo principal de este estudio fue determinar la relación entre las dimensiones del trabajo en equipo y las del desgaste. Si bien no se encontraron correlaciones clínica o estadísticamente significativas entre los puntajes totales del T-TPQ-S con los del CBI, entre las dimensiones sí se encontraron algunas correlaciones inversas, moderadas y estadísticamente significativas entre las subescalas de “unidad” y “desgaste relacionado con el trabajo”. Esto tiene sentido ya que se esperaría que el trabajo en equipo a nivel de unidad ayude a reducir los niveles de agotamiento que se experimentan en el ámbito laboral, pero sin impactar tanto en el nivel general de agotamiento percibido (desgaste personal) o en el asociado con la atención de los pacientes (desgaste relacionado con los usuarios). Cabe aclarar que estos niveles de desgaste no tienen relación con el apoyo, comunicación o liderazgo que se pueda tener en la unidad.

En la literatura existente se han reportado hallazgos similares que respaldan esta relación moderada. Por ejemplo, Al Sabei *et al.* documentaron una correlación ligeramente menor entre el trabajo en equipo y el desgaste (-0,26; p ≤ 0,001), en un estudio centrado en enfermeras ^47^. Además, Mijakoski *et al*. exploraron la posibilidad de que el trabajo en equipo actuara como mediador en la relación entre el compromiso y la satisfacción laboral ^48^, y encontraron que el trabajo en equipo potenciaba el efecto positivo del compromiso sobre la satisfacción laboral, aunque no moderaba la relación entre las demandas laborales y el agotamiento.

En una revisión sistemática, Welp y Manser analizaron 25 estudios que investigaban la relación entre el trabajo en equipo y el bienestar ocupacional de los médicos ^49^. Encontraron que la mayoría (77 %) de estos estudios reportaba asociaciones significativas entre estas variables. De estos, el 31 % indicaba una correlación positiva entre el trabajo en equipo y aspectos positivos del bienestar -como el compromiso laboral-, mientras que el 69 % señalaba una correlación negativa entre los indicadores positivos de trabajo en equipo y aspectos negativos del bienestar, como el desgaste.

En resumen, estos hallazgos sugieren que una percepción mejorada de la calidad del trabajo en equipo puede estar asociada con un mejor bienestar ocupacional y una reducción del agotamiento percibido. Además, estos resultados subrayan la necesidad de una exploración más profunda para entender completamente las dinámicas subyacentes que influencian estas relaciones y cómo podrían ser abordadas para mejorar la salud ocupacional y la seguridad del paciente en el entorno clínico.

Es importante señalar que la magnitud de la correlación identificada en el presente estudio es menor que la reportada en algunos trabajos anteriores. Una explicación plausible para esta discrepancia podría ser el posible sesgo en la medición del desgaste en esta investigación, lo que podría estar atenuando la verdadera magnitud de esta asociación. No obstante, este hallazgo subraya la necesidad de continuar investigando las dinámicas del trabajo en equipo y el desgaste profesional con un enfoque que permita comprender mejor cómo los factores individuales y organizacionales pueden influir en el reporte de estas variables y en la magnitud de estas relaciones.

Una limitación de este estudio radica en la tasa de respuesta alcanzada, que fue del 44 %, muy inferior al 80 % esperado. Esta discrepancia plantea preguntas críticas sobre la validez interna y externa de la investigación, sugiriendo la posibilidad de un sesgo de selección. Es plausible que los profesionales de la salud que optaron por no participar presenten diferencias sistemáticas significativas en comparación con aquellos que participaron. Específicamente, si los individuos con un mayor índice de desgaste profesional fueron menos propensos a participar, esto podría introducir un sesgo de selección significativo. Este sesgo podría haber resultado en una subestimación de las correlaciones reales entre el trabajo en equipo y el desgaste profesional, así como de las condiciones laborales en el hospital. La literatura epidemiológica indica que este tipo de sesgos pueden alterar de manera significativa las estimaciones de las relaciones entre variables, y por lo tanto, ocultar potenciales asociaciones significativas ^50^.

Este desafío metodológico no es ajeno a la literatura existente. Alahmari *et al.* reconocen la dificultad inherente a estudios que abordan el agotamiento en el personal de salud, ya que la tasa de respuesta a estas investigaciones tiende a ser invariablemente baja ^41^. La presente investigación enfrentó una complejidad adicional ya que anteriormente se había llevado a cabo una evaluación del desgaste profesional en la institución, en la que los participantes con resultados altos fueron convocados a la oficina de recursos humanos. Esta situación previa puede haber motivado a algunos profesionales de la salud a subestimar su nivel de agotamiento en el estudio actual, en un intento por eludir posibles consecuencias laborales. Aunque la literatura existente tiende a sobreestimar el desgaste profesional en los profesionales de la salud, en esta investigación se sospecha que el sesgo pudo haber operado en la dirección contraria.

En este contexto, es posible que la magnitud de las relaciones identificadas entre el trabajo en equipo y el desgaste profesional pueda estar subestimada. Es decir, estas relaciones podrían ser más fuertes de lo determinado en esta investigación, lo que resalta la necesidad de futuros estudios que puedan abordar estas limitaciones.

En cuanto a la validez externa, la capacidad para generalizar estos hallazgos a la población total de trabajadores de la salud en la institución podría cuestionarse. Los participantes podrían no ser representativos, lo que limitaría la aplicabilidad de las conclusiones ^51^. Finalmente, desde una perspectiva metodológica y ética, esta baja tasa de respuesta deja ver la poca disposición que podría tener el personal del hospital para responder este tipo de encuestas relacionadas con agotamiento, pues usualmente suele participar en diversos proyectos de investigación. Por lo tanto, dicho sesgo podría impactar en la utilidad de estos datos para la toma de decisiones a nivel institucional ^52^.

Se ha identificado una relación significativa, aunque moderada a débil, entre la percepción del trabajo en equipo y el desgaste profesional en el trabajo. Estos hallazgos sugieren que una mayor colaboración y un ambiente de apoyo pueden reducir el agotamiento laboral, mejorando así la seguridad del paciente.

Es importante reconocer que la relación es bidireccional: individuos con un nivel alto de desgaste profesional tienen más dificultades para colaborar efectivamente, lo que puede deteriorar la calidad del trabajo en equipo, generar una percepción negativa del ambiente laboral y, a su vez, exacerbar los niveles de desgaste.

Este enfoque integral confirma y amplía investigaciones previas, y subraya la necesidad de estrategias que aborden ambos aspectos de manera conjunta.

Los resultados de este estudio no deben generalizarse debido a la metodología empleada. Se recomienda realizar más investigaciones en el país para profundizar en este campo del conocimiento.

## Archivos suplementarios

**Tabla suplementaria 1.** Cuestionario de percepción del trabajo en equipo, versión en español (*TeamSTEPPS-S*)

		1	2	3	4	5
No. Afirmación		Totalmente en desacuerdo	En desacuerdo	Neutral	De acuerdo	Totalmente de acuerdo
Estructura del equipo						
1	Las habilidades del personal se distribuyen lo suficiente para que el trabajo pueda ser compartido cuando sea necesario.					
2	El personal se hace responsable de sus acciones.					
3	La información importante para la atención de los pacientes se comparte de forma oportuna entre el personal.					
4	En mi unidad se hace un uso eficiente de los recursos (por ejemplo, personal, suministros, equipos, información).					
5	El personal comprende sus roles y responsabilidades.					
6	En mi unidad las funciones de todos están interrelacionadas.					
7	En mi unidad se aprovechan bien los recursos para cumplir los objetivos.					
Liderazgo						
8	Los aportes del personal son considerados por el coordinador para tomar decisiones sobre la atención a los pacientes.					
9	El líder del equipo brinda espacios para discutir el desempeño del equipo luego de un incidente o evento adverso.					
10	El líder del equipo se toma el tiempo para reunirse con el personal y desarrollar un plan de atención a los pacientes.					
11	El líder de la unidad se asegura de que estén disponibles los recursos adecuados (por ejemplo, personal, suministros, equipos, información).					
12	El líder del equipo resuelve los conflictos adecuadamente.					
13	El líder del equipo es modelo de comportamiento apropiado de trabajo en equipo.					
14	El líder de la unidad se asegura de que el personal esté al tanto de cualquier situación o cambio que pueda afectar la atención de los pacientes.					
Conciencia situacional						
15	Los miembros del equipo se anticipan de forma efectiva a las necesidades de los demás miembros de su equipo.					
16	El personal está atento a la ejecución de las tareas de los demás miembros de su equipo.					
17	El personal comunica información nueva o importante de forma oportuna.					
18	El personal observa continuamente el entorno en busca de información importante para la atención de los pacientes.					
19	El personal comunica las eventualidades del servicio (por ejemplo, cambios de pacientes, disponibilidad de camas, daño de equipos).					
20	El personal se reúne para reevaluar los objetivos de atención de los pacientes cuando la situación ha cambiado.					
21	El personal ayuda a corregir los errores de cada uno para garantizar que los procedimientos se sigan correctamente.					
Apoyo mutuo						
22	El personal ayuda a sus compañeros cuando hay alta carga de trabajo.					
23	El personal solicita ayuda a sus compañeros cuando se sienten sobrecargados de trabajo					
24	El personal comunica a los demás las situaciones potencialmente peligrosas.					
25	La retroalimentación entre el personal se da de una manera que promueve interacciones positivas y cambios futuros.					
26	El personal aboga por los pacientes, incluso cuando su opinión entra en conflicto con la de un miembro con mayor jerarquía en el equipo.					
27	Cuando el personal está preocupado por la seguridad de los pacientes, cuestionan a otros hasta que están seguros de que la preocupación ha sido escuchada					
28	En el equipo se resuelven los conflictos, incluso cuando estos se hayan vuelto personales.					
Comunicación						
29	La información sobre la atención se explica a los pacientes y sus familias en términos sencillos.					
30	El personal se transmite información relevante de manera oportuna.					
31	Al comunicarse con los pacientes, el personal da suficiente tiempo para que hagan preguntas.					
32	Cuando se comunican entre sí, el personal usa terminología conocida por todo el equipo.					
33	El personal repite la información recibida verbalmente para verificarla.					
34	El personal tiene un protocolo estandarizado para intercambiar la información cuando se hace la entrega de los pacientes.					
35	El personal busca información de los pacientes de todas las fuentes disponibles.					
36	En la unidad se cuenta con canales de comunicación digitales o físicos que permitan el flujo de información de manera oportuna.					

El siguiente cuestionario pretende evaluar la percepción que usted tiene sobre algunos aspectos del trabajo en equipo en su unidad de trabajo. Por favor, lea las siguientes afirmaciones y marque con una equis (X) en la casilla que corresponda según su nivel de acuerdo. Para hacerlo piense en la forma como usted y sus compañeros de unidad han trabajado la mayor parte de las veces durante el último mes. Por favor, marque solo una respuesta para cada afirmación.

**Tabla suplementaria 2.** *Copenhagen Burnout Inventory (CBI)*

		0	1	2	3	4
No.	Afirmación	Nunca	Solo alguna vez	Algunas veces	Muchas veces	Siempre
1	¿Con qué frecuencia se siente cansado?					
2	¿Con qué frecuencia está físicamente agotado?					
3	¿Con qué frecuencia está psicológicamente agotado?					
4	¿Con qué frecuencia piensa “no puedo más”?					
5	¿Con qué frecuencia se siente agotado?					
6	¿Con qué frecuencia se siente débil y susceptible de enfermar?					
7	¿Su trabajo es emocionalmente agotador?					
8	¿Se siente agotado por su trabajo?					
9	¿Se siente frustrado por su trabajo?					
10	¿Se siente agotado al final de la jornada laboral?					
11	Por la mañana ¿le agota pensar en otro día de trabajo?					
12	¿Siente que cada hora de trabajo es agotadora?					
13	¿Tiene suficiente energía para la familia y los amigos durante el tiempo libre?					
14	¿Es duro trabajar con usuarios?					
15	¿Es frustrante trabajar con usuarios?					
16	¿Trabajar con usuarios consume su energía?					
17	¿Siente que da más de lo que recibe cuando trabaja con usuarios?					
18	¿Está cansado de trabajar con usuarios?					
19	¿A veces se pregunta cuánto tiempo podrá continuar trabajando con usuarios?					

A continuación, encontrará algunas afirmaciones referentes a sus sentimientos e ideas sobre su trabajo y de las consecuencias que tiene para usted como profesional y como persona. Indique con qué frecuencia siente o piensa lo que se describe en cada una de ellas.

Para responder marque con una X en la casilla que más se ajuste a su situación, así: 0: nunca; 1: solo alguna vez; 2: algunas veces; 3: muchas veces; 4: siempre.
